# Flooding Impairs Fe Uptake and Distribution in *Citrus* Due to the Strong Down-Regulation of Genes Involved in Strategy I Responses to Fe Deficiency in Roots

**DOI:** 10.1371/journal.pone.0123644

**Published:** 2015-04-21

**Authors:** Mary-Rus Martínez-Cuenca, Ana Quiñones, Eduardo Primo-Millo, M. Ángeles Forner-Giner

**Affiliations:** Department of Citriculture and Vegetal Production, Valencian Institute of Agrarian Research, Moncada, Valencia, Spain; South China Agricultural University, CHINA

## Abstract

This work determines the ffects of long-term anoxia conditions—21 days—on Strategy I responses to iron (Fe) deficiency in *Citrus* and its impact on Fe uptake and distribution. The study was carried out in *Citrus aurantium* L. seedlings grown under flooding conditions (S) and in both the presence (+Fe) and absence of Fe (-Fe) in nutritive solution. The results revealed a strong down-regulation (more than 65%) of genes *HA1* and *FRO2* coding for enzymes proton-ATPase and Ferric-Chelate Reductase (FC-R), respectively, in –FeS plants when compared with –Fe ones. H+-extrusion and FC-R activity analyses confirmed the genetic results, indicating that flooding stress markedly repressed acidification and reduction responses to Fe deficiency (3.1- and 2.0-fold, respectively). Waterlogging reduced by half Fe concentration in +FeS roots, which led to 30% up-regulation of Fe transporter *IRT1*, although this effect was unable to improve Fe absorption. Consequently, flooding inhibited 57Fe uptake in +Fe and –Fe seedlings (29.8 and 66.2%, respectively) and ^57^Fe distribution to aerial part (30.6 and 72.3%, respectively). This evidences that the synergistic action of both enzymes H+-ATPase and FC-R is the preferential regulator of the Fe acquisition system under flooding conditions and, hence, their inactivation implies a limiting factor of citrus in their Fe-deficiency tolerance in waterlogged soils.

## Introduction

As an essential micronutrient for plants, iron (Fe) participates in fundamental life sustaining processes. Despite its abundance in many cultivated soils, its acquisition by crop plants is often impaired by certain soil properties, e.g., alkaline pH or high bicarbonate content [1]. *Citrus*, like other dicotyledonous species, presents Strategy I responses to Fe-deficiency [2,3,4], including: (a) enhanced proton extrusion into the rhizosphere, which lowers the soil solution pH and increases Fe^3+^ solubilisation through the activation of a specific H^+^- ATPase [5]; (b) increased capacity to reduce ferric (Fe^3+^) to ferrous (Fe^2+^) forms [6] mediated by the ferric chelate reductase (FC-R) enzyme [7,8]; and, (c) enhanced Fe^2+^ uptake ability across the root cell membranes associated with the activation of a specific iron-regulated transporter (IRT) [9,10,11].

Genomic tools have contributed to a better understanding of the molecular and metabolic processes leading to Fe uptake in plants. With respect to proton release, some genes coding for Fe-regulated H^+^-ATPases have been characterized [5]. Thus, in citrus roots, *HA1* gene was induced in Fe-deficient roots, while *HA2* did not respond to Fe-deficiency [4]. In the other hand, reduction response is encoded by *FRO* gene family and the main genes have been identified in several species [7]. *FRO2*, which is expressed in the epidermal cells of roots, is believed to be primarily responsible for enhancing FC-R activity due to Fe-deficiency and its overexpression confers tolerance to low Fe conditions [4,8]. Finally, IRT genes code for family members of zinc transporter proteins (*ZIP*) in *Arabidopsis* [9,11]. Among them, *IRT1* gene is localized to the plasma membrane of epidermal cells inroots and its expression is induced by Fe-deficiency, generating the major transporter responsible for Fe uptake from the soil [4,10,11]. Recent works have focussed on characterising the response of the Fe- acquisition system in different citrus species and several inductive conditions of Fe-chlorosis; e.g, high contents of calcium carbonate, bicarbonate or metal cations in culture media [4,12,13,14,15]. However, very little knowledge about the influence of waterlogging conditions on Fe-chlorosis affection in citrus and in other plants is available.

Soil flooding has been widely reported to affect large areas of the world, generally in relation to poor soil drainage combined with excessive rainfall or irrigation. One major constraint deriving from excess water is the progressive reduction in both the soil O_2_ concentration and redox potential, which leads to the formation of reduced compounds of either chemical or biochemical origin. Soil alterations under these conditions have been widely reported [16,17]. Accordingly, flooding effects on plants are related mainly to declining aerobic root respiration, which impairs ATP synthesis, which in turn disturbs the plant metabolism. Moreover, soil flooding induces a variety of physiological disturbances that alter plant growth [18], including reductions in water flux from roots, hormonal imbalances, altered carbohydrate distribution, deficient nutrient uptake, early leaf senescence and injury in organs, which sometimes precede plant death [19].

Although the response is variable among species and cultivars, *Citrus* is considered a flooding-sensitive crop that it responds to waterlogging by restricting stomatal conductance to prevent water loss [20,21]. This fact appears to be hormone-regulated and is associated with abscisic acid accumulating in leaves, which induces stomatal closure [22]. Under these conditions, net CO_2_ assimilation by leaves subsequently reduces [20,23], which leads to altered carbohydrate distribution [24]. Impairment of the photosynthetic system may also generate excess reactive oxygen species (ROS) to result in oxidative damage to cells. It has been reported that coordinated antioxidant responses, involving the increased activity of superoxide dismutase and catalase, together with a modulation of the ascorbate-glutathione cycle, allow plants to cope with flooding-induced oxidative stress to a certain extent [25,26]. During prolonged soil flooding periods, reduced root hydraulic conductance [21,27,28] impairs water uptake, which causes leaf wilting and chlorosis [25]. Furthermore, the root signals and sensory mechanisms that trigger citrus responses to flooding have been recently described [21,22].

As a result of the root physiology dysfunction, flooding alters nutrient uptake and, therefore, the endogenous concentrations of macro- and microelements can be modified. Thus deficiencies in mineral elements, nutritional imbalances or changes in nutrient partitioning may appear in waterlogged plants, depending on plant species and soil type [29]. It has been reported that flooding alters nitrogen (N) pools and their partitioning in citrus as a result of reduced uptake and transport [24]. Moreover, in anaerobic soils, N may also be lost through denitrification processes [16], which occur because NO_3_^-^ is the first electron acceptor to be reduced following O_2_ depletion [30]. Additionally, waterlogging also prevents potasium (K) uptake and, therefore, lowers K concentrations in leaves [20,31], whereas it helps to other elements uptake by roots, such as copper and manganese [31].

Regarding Fe nutrition, anoxia conditions for several hours in flooded soils (short-term) promotes the reduction of Fe^3+^ to Fe^2+^ as a result of a lower soil redox potential [32,33]. Under these terms, excess uncontrolled Fe^2+^ uptake in acidic soils leads to very high Fe concentrations in plant tissues [16], which present toxicity symptoms, such as blackened root tips, inhibited root growth, and necrotic spots on leaves commonly known as bronzing [34]. These effects are attributed mainly to the oxygen stress induced by free radical generation [35]. In the other hand, long-term anoxia in the root zone leads to diminished Fe uptake [33]. A widely accepted theory to explain this effect is that in soils maintained during a long period of time under O_2_ deprivation conditions, CO_2_ concentration increases as a result of partially aerobic respiration and/or fermentation by roots, soil bacteria and fungi. When lime soils are flooded, the increased CO_2_ concentration combines with CaCO_3_ and H_2_O to form additional HCO_3_^-^, which inhibits Fe uptake by plants, and consequently leads to Fe chlorosis [14,36,37,38].

Further research is needed to provide insights into the intrinsic factors that limit Fe uptake by citrus roots under flooding conditions, and even in the absence of bicarbonate ions. For this purpose, we studied the effects of long-term anoxia conditions on Strategy I responses to Fe-deficiency in sour orange seedlings (*Citrus aurantium* L.) and the subsequent impact on Fe uptake and distribution in plants.

## Materials and Methods

### Plant material and pre-conditioning

*Citrus aurantium* (L.) seeds were germinated in a glasshouse using a sterile substrate comprising peat, coconut fibre, sand and perlite (50:25:20:5) supplemented with 1.38 g kg^-1^ calcium superphosphate. They were irrigated twice weekly with the following nutrient solution: 1.5 mM Ca(NO_3_)_2_, 1.5 mM KNO_3_, 1 mM MgSO_4_, 1.2 mM H_3_PO_4_, 20 μM Fe-EDDHA, 23.2 μM H_3_BO_3_, 27.2 μM MnSO_4_·H_2_O, 3.8 μM ZnSO_4_·7H_2_O, 0.27 μM MoO_3_ and 0.25 μM CuSO_4_·5H_2_O. The nutrient solution pH was adjusted to 6.0 with 1 M KOH or 1 M H_2_SO_4_ [4,33].

After 4 months, seedlings were selected based on uniformity of size and transplanted individually to opaque plastic 500-mL pots filled with coarse sand. Seedlings were then separated into two groups and fed with the above nutrient solution at a 2-fold strength and pH 7.5 either with or without 20 μM Fe-EDDHA (plants +Fe and -Fe, respectively). Seedlings were grown for 2 weeks under glasshouse conditions (16-h photoperiod, 250 μmol photons m^-2^ s^-1^ photosynthetic photon flux density, 400–700 nm, 16–18/26–28°C night/day temperatures and 80% relative humidity, RH).

### Induction of flooding stress

After pre-conditioning, plants from each group of the Fe-nutrition state (plants +Fe and -Fe) were divided into two subgroups. One subgroup of each Fe-state was well irrigated (Ct: control plants) 3 times/week with 200 mL of its corresponding nutrient solution either with or without 20 μM Fe-EDDHA (+FeCt and -FeCt, respectively). Any excess solution was drained out of the pot to therefore avoid salt from accumulating in sand. The other subgroups were introduced into separate plastic water tanks (49 x 39 x 14 cm) and underwent flooding stress treatments (S) by submerging pots in their corresponding nutrient solution either with or without 20 μM Fe-EDDHA (+FeS or -FeS, respectively). Whenever necessary, the nutritive solution was supplemented to maintain the water level 4 cm above the sand surface. An opaque plastic sheet was used to cover the container surfaces to avoid algal proliferation. Plants were maintained under the same previously described glasshouse conditions for 3 weeks. Then the seedlings from each Fe-state and treatment were carefully removed from the pots, and roots were washed with tap water to eliminate sand. Finally, whole seedlings were rinsed with de-ionised water before being processed for further measurements.

### Plant growth

Six seedlings per Fe-state and water stress treatment were separated into leaves, stems and roots, and fresh weight was determined. Organs were dried in a forced-draft oven at 70°C for 48 h and were re-weighed to record plant dry weight (DW, in g).

### Photosynthetic parameters

#### Chlorophyll concentration

The leaf chlorophyll (Chl) concentration per area was measured spectrophotometrically (*Lambda 25*, *PerkinElmer*, Shelton, CT, USA) according to Moran and Porath [39]. Leaf disks were cut with a calibrated cork borer (∅ = 7cm), incubated in 6 mL n,n-dimethylformamide at 4°C for 24 h and centrifuged for 15 min at 6,000 g and 4°C. The supernatant was left for 1 h in the presence of Na_2_SO_4_ and absorbance was measured at 664 nm and 647 nm. Measurements were taken on the two youngest fully expanded leaves of six seedlings per Fe-state and flooding treatment. The average value of the two leaves was considered to be representative of each individual plant.

#### Photosynthetic activity

The net CO_2_ assimilation rate (A_CO2_) of single attached leaves was measured outdoors between 10:00 am and 11:30 am on a sunny day, which allowed measurements to be taken under relatively stable conditions. Photosynthetically active radiation (PAR) on the leaf surface was adjusted to a photon flux density of 1,000 μmol photons m^-2^ s^-1^. A closed gas exchange (*CIRAS-2*, *PP-systems*, Hitchin, UK) was used for the measurements. Leaf laminae were fully enclosed within a PLC 6 (U) universal leaf autocuvette in a closed circuit model and were kept at 25±0.5°C with a leaf-to-air vapour deficit of about 1.7 Pa. The air flow rate through the cuvette was 500–1,500 mL min^-1^. Ten consecutive measurements were taken at 3-second intervals. Measurements were taken on the two youngest fully expanded leaves of all six seedlings per Fe-state and flooding treatment. The average value of the two leaves was considered to be representative of each individual plant.

### Total Fe concentration

Six seedlings per Fe-state and flooding treatment were separated into leaves, stems and roots, and were rinsed in distilled water containing a non-ionic detergent and finally 3 times in distilled water. Organs were dried in a forced-draft oven at 70°C for 48 h and dry weight was determined. Then samples were ground into a fine powder using a laboratory ball mill (Retsch MM301, Haan, Germany). Dried tissues (0.5 g) were burnt for 12 h in a muffle furnace at 550°C. Fe was extracted with 2% v/v nitric acid (Hiperpur Panreac, Fe<1 ppb) in an ultrasonic bath (*Fungilab S*.*A*., Sant Feliu de Llobregat, Barcelona, Spain) at 40°C for 30 min to obtain a final volume of 50 mL. All the analyses were carried out with ultrapure water (*Ultra Pure Water Systems Milli Q Plus*). The total Fe concentration ([Fe]_t_, in μg g^-1^ DW) was recorded by atomic absorption (*Aanalyst200*, *Perkin Elmer*, *Waltham*, Massachusetts, USA).

### ^57^Fe-labelling treatment

To determine the effect of different 3-week treatments (+Fe, -Fe, +FeS and -FeS) on the Fe uptake and transport capacity of each group of seedlings, these were exposed to nutrient solution to which a stable isotope ^57^Fe was added (95.6% atom ^57^Fe excess, Cambridge Isotope Laboratories, Inc., Andover, MA, USA) and ^57^Fe enrichment was measured in different plant organs (leaves, stems and roots). For this purpose, immediately after treatments, three sets of six seedlings per Fe-state and flooding treatment were placed for 24 h in bakers with 250 mL of-Fe nutrient solution at pH 7.5 and were supplemented with o,o-^57^FeEDDHA to reach a final concentration of 100 μM. To guarantee anoxia air conditions in the labelling solutions, the incubation media of the waterlogged plants were bubbled with nitrogen. The control solutions were bubbled instead with oxygen to simulate normal air conditions. This assay was performed in a controlled environmental chamber (*Sanyo MCR-350H*, *Sanyo Electric Biochemical Co*., Japan) under continuous light (200 μmol m^-2^ s^-1^, 400–700 nm) at 28°C and 80% RH. After labelling, extracellular ^57^Fe (from cell walls and apoplast) was pulled to obtain an accurate ^57^Fe concentration measurement in roots [40]. To this end, plants were maintained for 5 min in a desorption solution, comprising 5 mM MES-NaOH (pH 5.0), 5 mM CaCl_2_, 5 mM sodium ascorbate and 1 mM FeSO_4_. Sodium ascorbate and FeSO_4_ were added immediately prior to use and the desorption solution was readjusted to pH 5.0.

Then plants were separated into leaves, stems and roots, and were rinsed in distilled water containing a non-ionic detergent and finally 3 times in distilled water. Organs were dried in a forced-draft oven at 70°C for 48 h and dry weight was determined. Then samples were ground into a fine powder using a laboratory ball mill (Retsch MM301, Haan, Germany). Dried tissues (0.5 g) were burnt for 12 h in a muffle furnace at 550°C. Fe was extracted with 2% v/v nitric acid (Hiperpur Panreac, Fe<1 ppb) in an ultrasonic bath (*Fungilab S*.*A*., Sant Feliu de Llobregat, Barcelona, Spain) at 40°C for 30 min to a final volume of 50 mL. ^57^Fe enrichment (Δ^57^Fe, ^57^Fe in excess, as a %) was measured by multiple collector inductively coupled plasma mass spectrometry of high mass resolution (*MC-ICPMS*, *Thermo Finnigan Neptune*, Delaware, USA). Finally, the concentration of ^57^Fe in excess ([^57^Fe]_e_, in μg ^57^Fe g^-1^ DW) and the content of ^57^Fe in excess (^57^Fe_e_, in μg ^57^Fe) were determined [14].

### Root RNA extraction and real-time RT-PCR analysis

Gene expression was determined in the root tips of plants +Fe, -Fe, +FeS and -FeS. Immediately after treatments, the roots of three sets of six seedlings per Fe-state and flooding treatment were rinsed 3 times in distilled water, frozen at -80°C and nitrogen-powered. Total RNA was extracted from approximately 0.5 g of frozen root tissue using the RNeasy Plant Mini Kit (Qiagen, Hilden, Germany). RNA samples were treated with RNase-free DNase (Qiagen) through column purification following the manufacturer’s instructions. RNA quality (OD_260_/OD_280_ ratio) and concentration were determined spectrophotometrically (Nanodrop Technologies, Thermo Fisher Scientific, Delaware, USA). A quantitative real-time reverse transcription polymerase chain reaction (RT-PCR) was run in a LightCycler 2.0 Instrument (Roche, Mannheim, Germany), equipped with the Light Cycler Software, version 4.0. Reactions contained 2.5 units of MultiScribe Reverse Transcriptase (Applied Biosystems, Roche Molecular Systems, New Jersey, USA), 1 unit of RNase Inhibitor (Applied Biosystems), 2 μL LC Fast Start DNA Master PLUS SYBR Green I (Roche Diagnostics GmbH, Mannheim, Germany), 25 ng of total RNA and 0.250 μM of the specific forward and reverse primers of each gene in a total volume of 10 μL. Incubations were carried out at 48°C for 30 min, 95°C for 10 min, followed by 45 cycles at 95°C for 2 s, 58°C for 8 s and 72°C for 8 s. The fluorescent intensity data were acquired during the 72°C-extension step and were transformed into relative mRNA values using a 10-fold dilution series of an RNA sample as a standard curve. The relative mRNA levels were then normalised to total RNA amounts [41], and an expression value of 1 was arbitrarily assigned to the values of the +FeCt seedlings. Actin was used as the reference gene [42]. The specificity of the amplification reactions was assessed by post-amplification dissociation curves and by sequencing the reaction product. The resolution of the curve expressions was confirmed.

Putative genes were identified by a homology search with related genes from “Haploid Clementine Genome, International Citrus Genome Consortium, http://www.phytozome.net/clementine” [43]. Synthetic oligonucleotides were designed to amplify the gene from the selected clones and, as mentioned previously, were sequenced for confirmation. The specific primers for

- *HA1* F: 5’-GGACGCGTTTGGTGTAAGAT-3’
  R: 5’-GAAGTCCAGGGCGTTCAATA-3’
- *FRO2* F: 5’-GGAGGAGCCAAAACAAGATG-3’
  R: 5’-CAGCCAAGAAACACAGCAAA-3’
- *IRT1* F: 5’-CTCAGTTGGAGCCACAAACA-3’
  R: 5’-GTACTCCGCCTGAAGAATGC -3’

were used to amplify the fragments of the respective genes by qRT-PCR [4]. At least three independent RNA extractions per treatment and three RT-PCR reactions with three technical replicates per sample were performed.

### Acidification of root media

To quantitatively determine H^+^ extrusion, three sets of six seedlings per Fe-state and flooding treatment were transferred to beakers containing 250 mL of their corresponding nutrient solution, adjusted to pH 7.5 with 5 mM NaOH (Panreac, Barcelona, Spain). Beakers were completely covered with aluminium foil to exclude light and solutions were bubbled continuously with air. Plants were maintained in a controlled environment chamber (Sanyo MCR-350H, Sanyo Electric Biochemical Co, Japan) under continuous light (200 μmol m^-2^ s^-1^ photosynthetic photon flux density, 400–700 nm) at 28°C and 80% RH. Medium acidification was determined by measuring pH changes in the nutrient solution after an 8-hour incubation period (pHmeter, Consort C531, Turnhout, Belgium).

### Fe Chelate Reductase (FC-R) activity

FC-R activity was determined by measuring the formation of Fe^2+^ and the BPDS (bathophenanthroline-disulfonic acid disodium salt hydrate) complex from Fe^3+^-EDTA [44]. Tips of apical root segments (about 5–8 mm long) with a total fresh weight of 0.020 g from six seedlings per Fe-state and flooding treatment, were rinsed with 0.2 mM CaSO_4_·2H_2_O for 5 min. Then they were incubated in 10 mL of fresh nutrient solution (without Fe) supplemented with 0.3 mM BPDS (Acros Organics, New Jersey, USA) and 100 μM Fe^3+^-EDTA (Sigma-Aldrich, St Louis, MO, USA). The pH of the assay solution was previously adjusted to pH 5.5 with 5 mM MES-NaOH (morpholineethanesulfonic acid) (Panreac, Barcelona, Spain). The beaker was completely covered with aluminium foil to exclude light at 23°C. After an incubation period of 1–6 h, aliquots were removed hourly and absorbance at 535 nm was determined by a spectrophotometer (Mikrowin 2000, Asys, Eugendorf, Austria). BPDS forms a stable, water-soluble red complex with Fe^2+^ and only a weak complex with Fe^3+^. The amount of reduced Fe was calculated by the Fe^2+^-(BPDS)_3_ complex concentration by applying an extinction coefficient of 22.14 mM^-1^ cm^-1^, and was expressed in μmol Fe^2+^ reduced g^-1^ root FW h^-1^.

### Statistical analyses

The experiment was a completely randomised 2 x 2 factorial design with two Fe-state and two water treatments. The values of DW, chlorophyll content, A_CO2_, [Fe]_t_ concentration and FC-R activity were the mean of six independent plants per Fe-state and flooding treatment. The values of [^57^Fe]_e_ concentration, acidification of root media and RT-PCR analysis were the mean of three replications (six plants each) per Fe-state and flooding treatment. Data were submitted to an analysis of variance (ANOVA) with Statgraphics Plus, version 5.1 (Statistical Graphics, Englewood Cliffs, NJ, USA), prior to testing for normality and homogeneity. When the ANOVA showed a statistical effect, means were separated by multiple comparison procedure to determine which means are significantly different from which others using Fisher´s least significant differences (LSD) test at the 95% confidence level (P < 0.05). Within each row, treatments with same letter form a group of means within which there are no statistically significant differences.

## Results

### Plant growth

Three weeks after plants were exposed to flooding treatment, plants developed chlorosis symptoms in the apical leaves, and even more quickly in the plants submitted to Fe-deficiency. On a DW basis (Table 1), the treatment lacking Fe (-FeCt) lowered the biomass of leaves and stems, but not significantly that of roots when compared to the plants grown in Fe-supplied nutrient solution (+FeCt). After 21 days of waterlogging, only the DW of roots lowered in both plants +FeS and -FeS *vs*. their corresponding controls (plants +FeCt and -FeCt, respectively). However, no differences were observed in leaves and roots as a result of the flooding treatments. Finally, the combination of Fe-state and water stress significantly affected stems biomass.

**Table 1:** Dry weight (g), concentration of total Fe ([Fe]_t_), concentration of ^57^Fe in excess ([^57^Fe]_e_) and content of ^57^Fe in excess (^57^Fe_e_) in the leaves, stems and roots measured in *Citrus aurantium* seedlings.

	+Fe		-Fe		ANOVA		
	Ct	S	Ct	S	Fe	S	Fe x S
DW (g)							
Leaves	10.33a	10.04a	5.91b	5.87b	***	ns	ns
Stems	4.65a	4.25b	3.18c	3.00c	***	*	***
Roots	2.86a	2.07b	2.50a	1.81b	**	***	ns
[Fe]_t_ (μg Fe g^-1^ DW)							
Leaves	75.85a	35.87b	31.21b	25.61b	***	***	***
Stems	6.42a	4.20b	4.90b	4.25b	**	***	***
Roots	496.72a	248.08b	127.42c	114.83c	***	***	***
[^57^Fe]_e_ (μg ^57^Fe g^-1^ DW)							
Leaves	0.94b	0.68c	2.49a	0.70c	***	***	***
Stems	0.29b	0.20c	0.70a	0.17c	***	***	***
Roots	3.59b	2.52c	7.33a	2.48c	***	***	***
^57^Fe_e_ (μg ^57^Fe plant^-1^)							
Whole seedling	21.35b	12.89c	35.16a	9.13c	***	***	***
Aerial part	11.06b	7.68c	16.81a	4.65c	***	***	***
Roots	10.29b	5.21c	18.35a	4.48c	***	***	***

Plants were grown for 21 days in Fe-sufficient (+Fe) or Fe-deficient (-Fe) nutrient solutions with the non-stressed (Ct) or the flooding treatment (S). For ^57^Fe determinations, plants were labelled immediately after treatments with 100 μM o,o-^57^FeEDDHA for 24 h.

^1^Values for DW and [Fe_t_] are the means of six independent plants per treatment (n = 6). Values for [^57^Fe_e_] and ^57^Fe_e_ are the means of three independent experiments (n = 3). For a comparison of the means, an ANOVA followed by the Fisher´s least significant differences (LSD) test, calculated at the 95% confidence level, was performed. Different letters in the same row indicate significant differences between the different genotypes and treatments. Levels of significance are represented by *P <* 0.05 (*), *P <* 0.01 (**), *P <* 0.001 (***) and ns (non-significant).

### Chlorophyll concentration and photosynthetic activity

Fe deficiency markedly reduced the Chl a and b concentration values, which were 83.9% and 82.2% lower in-FeCt than in +FeCt respectively (Table 2). Waterlogging also affected the chlorophyll content in leaves since the Chl a and b concentrations in the +FeS seedlings lowered by 39.0% and 67.3%, respectively, when compared with the +FeCt ones. However, the-FeS seedlings showed only a 52.1% reduction in the Chl b concentration if compared to the-FeCt ones. The Chl a/b ratio was 1.9- and 1.7-fold higher in the leaves from +FeS and -FeS, respectively, when compared with those from their corresponding control seedlings. Both Chl concentrations were significantly different when both factors were analyzed together, although Chl a/b ratio was not. At least, the leaf net CO_2_ assimilation rate (A_CO2_) dropped by 2.2-fold in plants +FeS vs. the +FeCt ones, while no significant reduction was observed between the Fe-deprived plants. A_CO2_ was also significantly affected by the nutritional state of the plant and water stress.

**Table 2:** Chlorophyll (Chl) *a* and *b* contents, ratio *a/b* and gas exchange parameter (A_CO2_) measured in fully developed leaves of *Citrus aurantium* seedlings.

	+Fe		-Fe		ANOVA		
	Ct	S	Ct	S	Fe	S	Fe x S
Chl (μmol m^-2^)							
*a*	356.4a	217.5b	57.4c	46.9c	***	**	**
*b*	273.5a	89.4b	48.8c	23.4d	***	***	***
*a/b*	1.3c	2.4a	1.2c	2b	*	***	ns
A_CO2_ (μmol CO_2_ m^-2^ s^-1^)	6.8a	3.1b	2.5b	1.3c	**	**	*

Plants were grown for 21 days in Fe-sufficient (+Fe) or Fe-deficient (-Fe) nutrient solutions with the non-stressed (Ct) or the flooding treatment (S).

^1^Values are the means of six independent plants per treatment (n = 6). For a comparison of the means, an ANOVA followed by the Fisher´s least significant differences (LSD) test, calculated at the 95% confidence level, was performed. Different letters in the same row indicate significant differences between the different genotypes and treatments. Levels of significance were represented by *P <* 0.05 (*), *P <* 0.01 (**), *P <* 0.001 (***) and ns (non-significant).

### Iron concentration

The Fe-deprived (-FeCt) plants presented lower Fe concentration values in leaves, stems and roots than the Fe-supplied (+FeCt) plants (reductions of 58.8%, 23.8% and 74.3%, respectively; Table 1). Three weeks after the waterlogging treatment, the Fe concentration in the above organs was markedly reduced by flooding. Thus plants +FeS showed a markedly lower Fe concentration in organs (decreases of 52.7%, 34.5% and 50.0% in leaves, stems and roots, respectively) if compared to +FeCt organs. However, no significant effect of flooding on Fe concentration was observed between the organs of plants-FeS and -FeCt. Finally, the combination of Fe-state and water stress treatments significantly affected total Fe concentration in leaves, stems and roots.

### ^57^Fe uptake

As observed in ^57^Fe determinations (Table 1), the-FeCt seedlings presented the highest [^57^Fe_e_] values in all the plant organs (above 2-fold higher than in +FeCt in all the fractions). When this parameter was examined in the plants submitted to waterlogging, the data revealed that [^57^Fe]_e_ in the +FeS seedlings had lowered in all the plant organs (27.7%, 31.1% and 29.8% in leaves stems and roots, respectively) in comparison to the +FeCt seedlings. These reductions were much more marked in the Fe-starved plants since the [^57^Fe]_e_ in leaves, stems and roots were, respectively, 71.9%, 75.7% and 66.2% lower in the-FeS than in the-FeCt seedlings. Finally, +FeS and -FeS plants presented the lowest [^57^Fe]_e_ concentration in organs although no significant differences were found between them.

In aerated (Ct) plants, Fe-deficiency increased ^57^Fe_e_ uptake by 64.7% when compared with the Fe-sufficient plants. Flooding treatment reduced ^57^Fe_e_ absorption by 39.6% and 74.0% for seedlings +FeS and -FeS, respectively, in comparison to their corresponding controls. The amount of ^57^Fe_e_ transported to the aerial part of seedlings (leaves + stem) followed the same pattern as that of ^57^Fe_e_ absorbed by the whole plant. Thus the-FeCt seedlings accumulated 52.0% more ^57^Fe_e_ in the top part of the plant than the +FeCt ones, whereas flooding treatment reduced this parameter in seedlings +FeS and -FeS to 30.6% and 72.3%, respectively when compared with their corresponding controls. The combination of Fe-deprivation and flooding stress reduced ^57^Fe_e_ to 58.0% and 56.5% in the aerial part and roots, respectively.

### Acidification of media

**Fig 1:**
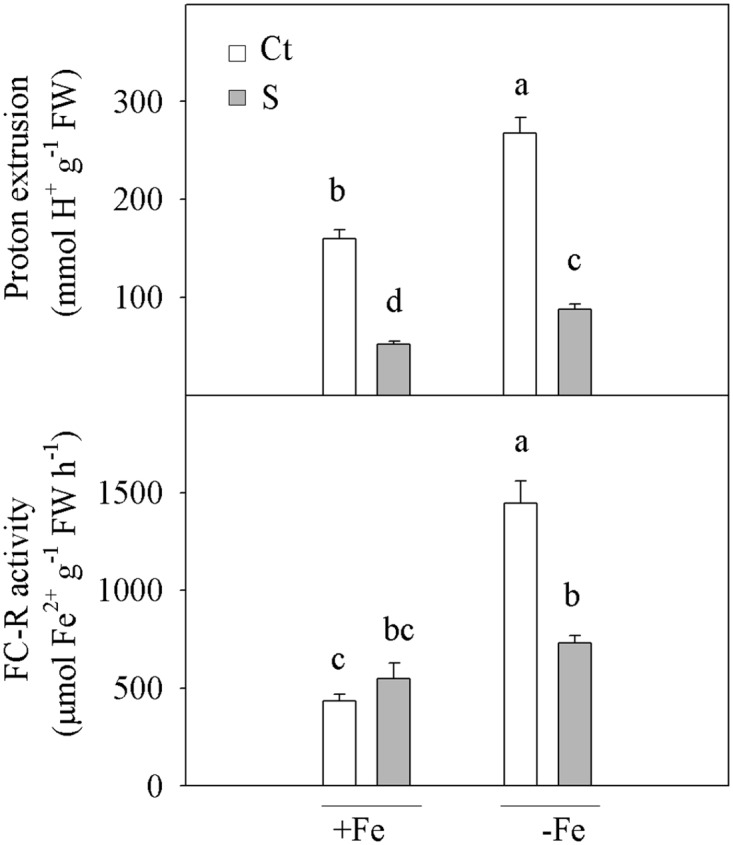
Acidification and Fe-reduction capacities in the roots. Acidification capacity measured as protons extruded for an 8-hour incubation period and Ferric-chelate reductase (FC-R) activity in the roots of *Citrus aurantium* seedlings. Plants were grown for 21 days in Fe-sufficient (+Fe) or Fe-deficient (-Fe) nutrient solutions with the non-stressed (Ct) or the flooding treatment (S). The values for proton extrusion and FC-R activity are the means of three (n = 3) and six (n = 6) independent experiments, respectively. For a comparison of the means, an ANOVA, followed by the LSD test, calculated at the 95% confidence level, was performed. Bars with different letters indicate significant differences at *P <0.05 using the LSD multiple range test.

When the plants were transferred to incubation media, H^+^-extrusion started immediately in all the plant groups, although successive solution pH measurements showed that the acidification of media occurred more rapidly in the controls than in the waterlogged plants (results not shown). After an 8-hour incubation, seedlings-FCt exhibited the highest H^+^-extrusion values (1.7-fold higher than +FeCt; Fig 1). Waterlogging similarly reduced the acidification capacity in both seedlings +FeS and -FeS (around 3.1-fold) when compared with their corresponding controls (+FeCt and -FeCt, respectively). Moreover, the interaction of both factors significantly altered H^+^ extrussion in the roots.

### FC-R activity

The Fe-deprived (-FeCt) plants presented the highest Fe^3+^-reducing activity in roots (3.3-fold higher than in +FeCt; Fig 1). However, waterlogging affected FC-R activity in each Fe-nutrition state differently. On the one hand, flooding in the Fe-supplied plants (+Fe) did not significantly alter reduction capacity, which remained at a low level. On the other hand, the amount of Fe^3+^ reduced to Fe^2+^ by roots from the -FeS seedlings diminished almost to half of that of those from -FeCt. Interestingly, -FeS and +FeS seedlings showed similar FC-R activities.

### Gene expression analysis

**Fig 2:**
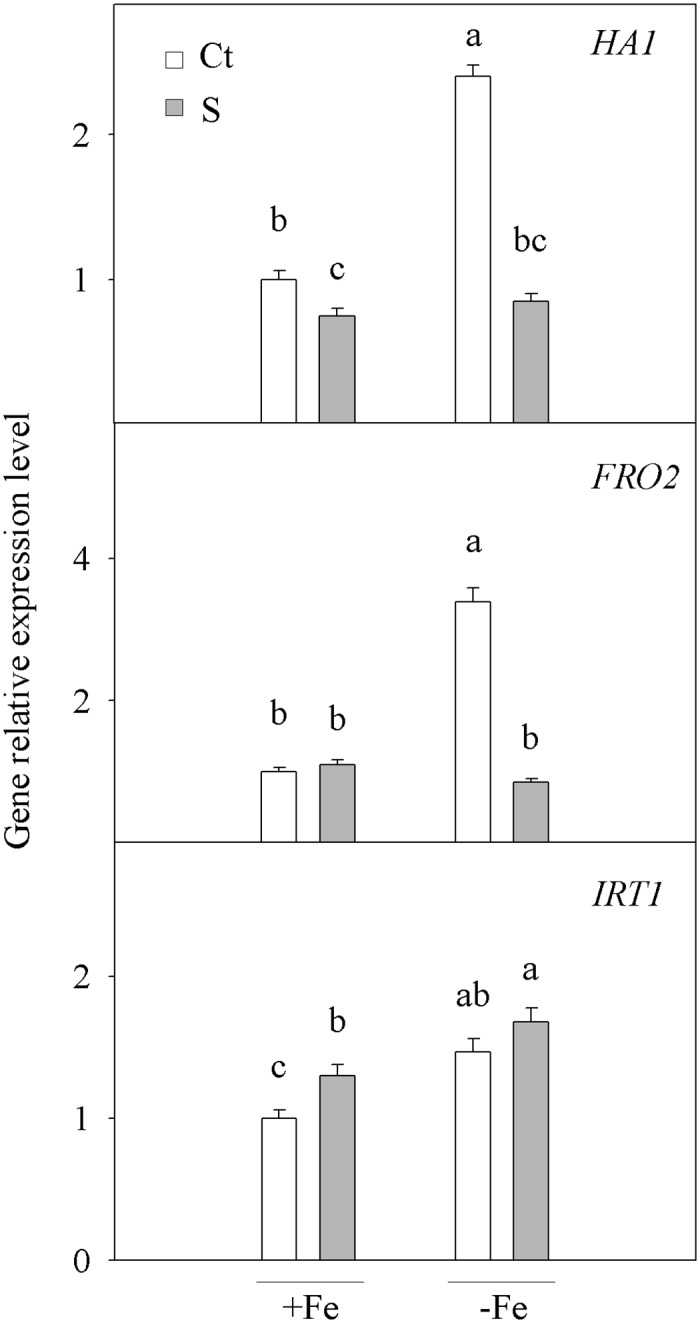
Gene relative expression level. Relative expression of genes *HA1*, *FRO2* and *IRT1* measured by a real-time RT-PCR analysis in the roots of *Citrus aurantium* seedlings. Plants were grown for 21 days in Fe-sufficient (+Fe) or Fe-deficient (-Fe) nutrient solutions with either the non-stressed (Ct) or the flooding treatment (S). Values are the means of three independent experiments (n = 3). For a comparison of the means, an ANOVA followed by the LSD test, calculated at the 95% confidence level, was performed. Bars with different letters indicate significant differences at *P <0.05 using the LSD multiple range test.

According to molecular study (Fig 2), plants-FeCt presented a marked increase in the abundance of the mRNA transcripts from the three genes (2.4-, 3.4- and 1.5-fold for *HA1*, *FRO2* and *IRT1*, respectively) compared with the +FeCt ones as a result of Fe-deficiency. The flooding conditions diminished *HA1* gene activity in seedlings +FeS and -FeS (1.3- and 2.8-fold, respectively) when compared with their corresponding controls (+FeCt and -FeCt, respectively; Fig 2). Interestingly, this decrement was specially important in-FeS seedlings which reached similar expression levels to +FeCt ones. The flooding treatments lowered the expression of gene *FRO2*, which regulates the FC-R enzyme, by 74.9% in the roots from-FeS compared to those from—FeCt, whereas the roots in +FeS and +FeCt remained at a similar level. Again, *FRO2* expression in -FeS seedlings was markedly reduced to +FeCt levels. Finally, the mRNA transcripts abundance of iron transporter gene *IRT1* was enhanced by flooding in the Fe-supplied plants roots, which was 1.3-fold higher in the roots of +FeS than in those of +FeCt. This effect was not observed in the Fe-starved plants (-FeS and -FeCt), where the *IRT1* expression levels remained high.

## Discussion

The data presented herein confirm that induced Fe-deficiency in citrus seedlings has a repressive effect on plant biomass, which is far more important in the shoot than in the root fraction. Identical results have been described for pea [45], pear and olive [46], peach [47] and *Arabidopsis thaliana* [48]. After plants were exposed to the 21-day flooding treatment, loss of root biomass occurred in both Fe-nutritional states. This effect has been observed in several crops and differences between genotypes suggest variations in their mechanism of response to flooding conditions [49]. In *Rumex*, it was found a direct relation between a reduced root growth rate and altered root porosity, which was closely explained by a differential internal oxygen supply to roots [50]. In this sense, aerenchyma and an inducible barrier to radial oxygen loss facilitates root aeration in wetland species [51,52].

Additionally, chlorosis symptoms rapidly developed in the apical leaves of the plants submitted to Fe-deficiency, and even more quickly with the waterlogging treatment. The study of pigment contents showed that Fe deprivation reduces leaf Chl, which is consistent with previous reports indicating that Fe-deficiency lowered the leaf Chl concentration in plants such as peach [53], grapevine [54], *A*. *thaliana* [48], pea [55] and citrus [13,14]. The effect of treatments enhancing Fe deficiency on Chl content has been ascribed to the role of Fe in the formation of some precursors of Chl biosynthesis [56]. Even more, flooding significantly lowered pigment contents, especially the Chl b level, more markedly in the +FeS plants, but also substantially in the-FeS plants. Accordingly, Ladygin [57] indicated an additive combined effect of Fe deficiency and root anoxia on the biochemical composition and structure of pea leaf chloroplast. The higher chl a/b ratio observed in the leaves of flooded plants assumed a faster degradation of Chl b than of Chl a. This is indicative of the advancing reduction of the light-harvesting chl a/b-protein complex in chloroplast membranes in comparison to the chl a-protein complexes of the reaction centres of PS I and II [57].

Fe-deprivation also suppressed A_CO2_ rate, and root anoxia significantly reduced this parameter in the leaves of plants +FeS and -FeS. A lower A_CO2_ value, due to Fe-deficient conditions, is a well-known response in citrus and other plant species [13,14,53,58]. In line with our findings, the suppression of A_CO2_ and intensification of dark respiration in leaves are characteristic root hypoxia features under optimum mineral nutrition conditions of plants [24,25,57]. The data in Table 2 indicate that the combined action of the disruption of Fe uptake and root anoxia caused a further drop in the photosynthesis rate by a factor of 5.2. This effect has been previously described in pea-flooded plants [57]. Accordingly, Fe deficiency and root anoxia develop different and independent action mechanisms on the leaf chloroplast structure and function, and their effects are additive when both stresses occur simultaneously [57].

Finally, typical responses under flooding conditions involve changes in the mineral element content in plants [18]. As expected, the Fe concentration of the citrus plants submitted to Fe-deficiency significantly reduced in all plant organs. Hence this, and also a long-term flooding treatment, caused the Fe concentration of all +FeS plants fractions to drastically lower. A previous report has demonstrated that exposure of other species to waterlogging stress markedly reduces the concentration of this element in plants [33,59]. This suggests that waterlogging stress promotes Fe deficiency in citrus, likely through the impairment of Fe uptake in plants. Therefore, the next step was to study the mechanisms whereby flooding limits Fe acquisition by citrus roots, and the regulation of Strategy I responses in the root system under these conditions.

Firstly, RT-PCR analysis indicated that flooding drastically disrupts the induction of *HA1* gene expression, despite the lower Fe nutritional state of plants after the waterlogging treatment when compared with their control plants. Consistently with this, the amount of H^+^ that flooded plants released to the media diminished, which suggests the inactivation of the plasma membrane H^+^-ATPase enzyme from root epidermal cells. Conversely, it is widely accepted that the enhanced acidification capacity of root rhizosphere is the first step response of roots to Fe-deficiency and to, therefore, improve the Fe-acquisition system against Fe-limiting conditions [60]. So the disruption of H^+^ extrusion to media by waterlogging prevents a transmembrane electrochemical gradient from being generated, which constitutes the driving force for Fe^2+^ uptake [61]. When roots suffer from prolonged O_2_ deprivation, mitochondrial respiration is inhibited and oxidative phosphorylation is interrupted, and both cause loss of energy in plants. Thus absence of electron acceptors leads to NAD(P)H accumulation and to reduced ATP production [62]. Although the energy flow is redirected through alternative anaerobic reactions, these processes (e.g., production of lactate, ethanol, etc.) lead to a very modest energy harvest. Under such conditions, the drop in the cytoplasmic ATP level is sharp and H^+^-pumps, major consumers of ATP, work at a very low activity rate, which results in a poorer H^+^ export across the plasma membrane [63]. Consequently, the resting potential in the plasma membrane diminishes and comes close to the diffusion potential, and H^+^ availability for symporters is drastically reduced. Hence, membrane depolarisation likely disturbs, and even inverts, the inward driving force for cations, and the diffusion potential might actually promote ions to leak out of the cell. Moreover, Fe may also be immobilised in the root apoplast, probably due to enhanced apoplasmic pH [4,14,64].

We also monitored the reduction capacity of the roots, reported as the second mechanism of response involved in the Fe-uptake system on the root surface [7]. The enhanced expression level of gene *FRO2* in the -FeCt plants presented herein confirms the inductive behaviour of enzyme FC-R as a result of Fe-deficiency, which indicates a strong Fe^3+^ reduction response in citrus roots [4] and other species [8,65]. However, the effect of the waterlogging treatment on the -FeS plants not only impaired the induction of gene *FRO2*, but also sharply lowered its expression level to similar values like those recorded in the +Fe plants. This suggests that the expression of gene *FRO2* under waterlogging conditions is not regulated by the plant’s Fe-nutritional state. In line with this, Lucena et al. [37] reported the inhibited expression of those genes that encode for enzyme FC-R in Fe-chlorotic plants induced by bicarbonate ion, probably through an altered expression of Fe efficiency reactions (*FER* or *FER*-like) transcription factors. Besides *FRO2* down-regulation, enzyme FC-R activity, measured in-FeS roots, strongly paralleled the gene’s behaviour pattern. However, it is noteworthy that other factors, such as lack of NADH deriving from oxygen depletion, likely exerts an adverse effect on FC-R activity. Absence of O_2_ for a short time period generates a more negative redox potential in roots, which favours the formation of NADH and NADPH [66,67], these being the main electron donors for the reduction of Fe^3+^ to Fe^2+^ [68]. This should favour FC-R activity in roots and should, therefore, increase Fe^2+^ availability to plants under short-term flooding conditions, as previously reported by Zude-Sasse and Schaffer [33]. However, a long-term O_2_ deficit enhances ROS production and, since the reactions to cope with cellular oxidative damage [69] need to consume electron donors [70,71], NADH availability for Fe-reduction activity in waterlogged plants is restricted.

A clear inverse relation also appears between the Fe-nutritional state of roots, measured by Fe concentration, and the expression level of gene *IRT1*, which has been demonstrated to encode a major transporter responsible for high-affinity metal uptake [10,11]. Thus the reduction to a half on the Fe concentration in +FeS roots increased gene *IRT1* activity in these roots by about 30%, whereas a 77% decrease in the Fe concentration in-FeS roots resulted in transcript level abundance, which increased by almost 60% when compared with +FeCt roots. This observation supports the key role of *IRT1* in the reaction of citrus roots to Fe-deficiency conditions by enhancing root Fe-transport capacity, just as previous reports have indicated [9,10,11,72]. However, induction of gene *IRT1* expression under waterlogged conditions was unable to improve Fe uptake by roots for it to reach the same level as in the Fe-deficient (-FeCt) plants.

The observations made above evidence that the synergistic action of both H^+^-ATPase and FC-R enzymes is the preferential regulator of the Fe acquisition system. Some authors have reported that in well aerated soils, Fe uptake by roots is inhibited when these were exposed to other stress conditions, which affects acidification and/or reduction capacity [4,14,38,46,55]. Impairment in the regulation of genes *HA1* and *FRO2* in waterlogging-stressed seedlings leads to the blockage of Fe uptake capacity, as supported by ^57^Fe labelling experiments. As expected, the lower Fe concentration recorded in -FeCt roots, in comparison to that in the +FeCt ones, resulted in the strongest ^57^Fe uptake after the labelling experiment given the induction of Strategy I responses [4,54,55]. Accordingly, Fox et al. [40] obtained and increase in Fe uptake induced by Fe-deficiency by using a ^59^Fe chelate on *Pisum sativum*. Furthermore, previous reports of Fe transport to the upper portions of *Citrus* plants carried out using ^55^Fe [3] and ^57^Fe [14], suggested that the ^55^Fe transported to the stems and leaves was proportional to Fe actively absorbed by roots [3], and it was related with the tolerance of genotypes under Fe-deficient conditions [3,4,14]. However in plants -FeS, no increase in ^57^Fe uptake was detected despite the low Fe concentration in roots (similar to that in-FeCt roots). Moreover, long-term flooding treatment also generated a lower Fe concentration in the roots of the Fe-supplied (+FeS) plants when compared with that of the +FeCt ones, although ^57^Fe uptake was lower in the latter than in the former. So the ^57^Fe uptake data indicate that regardless of the plant’s nutritional status, waterlogging markedly impairs Fe uptake by roots, being major causes of this constraint the inhibitory effects of anoxia on *HA1* and *FRO2* genes likely of the limited energy supply derived from the anaerobic metabolism, which may not suffice to maintain the Fe uptake process [4,14,64]. Consequently, ^57^Fe transport and distribution to the aerial part under flooding conditions was also markedly blocked. This impairment was previously observed in citrus seedlings induced to Fe-deficiency by the presence of ion bicarbonate [14]. However, the regulation mechanism differed from the results obtained under flooding conditions. In that case, although *HA1* and *FRO2* genes were up-regulated, ^57^Fe transport was reduced because bicarbonate because of the high amounts of Fe trapped in the apoplast of root cells, due to increased apoplast pH.

It is worth mentioning the particular case of lime alkaline soils if we consider their ability to induce Fe-deficiency in citrus and other species, especially under flooding conditions. It is here where bicarbonate production is enhanced as long-term waterlogging leads to oxygen depletion and CO_2_ accumulation due to a hindered gas exchange from soil solution to ambient air [33]. Thus it is well-known that a high bicarbonate level induces Fe deficiency in plants through buffering soil solution to inhibit FC-R induction, which enhances the alkalinisation of xylem sap and the cell apoplast, and prevents Fe uptake and transport from roots to shoots [14,38,73]. However, our experiment was performed in the absence of HCO_3_^-^, which indicates that inhibition of Fe uptake by flooding under field conditions is not due only to the presence of this ion.

Finally, it has been widely demonstrated that the citrus rootstocks which better tolerate Fe-deficiency are those that present clear Strategy I responses to enhanced H^+^-ATPase and FC-R activities in roots [2,3,4]. Flooding prevents these responses from developing in roots and, therefore, disables plants to react against low Fe levels in the medium, thus reducing their tolerance to Fe-deficiency. From the results presented herein, we conclude that long-term waterlogging markedly reduces Fe uptake in citrus because these conditions impair the responses of roots to Fe-deficiency, firstly through H^+^-ATPase inactivation and then by preventing FC-R activity from being induced.

## References

[1] Hell R, Stephan UW. Iron uptake, trafficking and homeostasis in plants. Planta 2003;216: 541–551. 12569395 10.1007/s00425-002-0920-4

[2] Treeby M, Uren N. Iron deficiency stress responses amongst citrus rootstocks. Z Pflanz Bodenk. 1993;156: 75–81.

[3] Manthey JA, McCoy DL, Crowley DE. Stimulation of rhizosphere iron reduction and uptake in response to iron deficiency in citrus rootstocks. Plant Physiol Bioch. 1994;32: 211–215.

[4] Martínez-Cuenca MR, Forner-Giner MA, Iglesias DJ, Primo-Millo E, Legaz F. Strategy I responses to Fe-deficiency of two *Citrus* rootstocks differing in their tolerance to iron chlorosis. Sci Hortic. 2013;153: 56–63.

[5] Santi S, Cesco S, Varanini Z, Pinton R. Two plasma membrane H^+^-ATPase genes are differentially expressed in iron-deficient cucumber plants. Plant Physiol Bioch. 2005;43: 287–292. 15854837 10.1016/j.plaphy.2005.02.007

[6] Kim SA, Guerinot ML. Mining iron: Iron uptake and transport in plants. FEBS Lett. 2007;581: 2273–2280. 17485078 10.1016/j.febslet.2007.04.043

[7] Jeong J, Connolly EL. Iron uptake mechanisms in plants: Functions of the FRO family of ferric reductases. Plant Sci. 2009;176: 709–714.

[8] Connolly EL, Campbell NH, Grotz N, Prichard CL, Guerinot ML. Over-expression of the *FRO2* ferric chelate reductase confers tolerance to growth on low iron and uncovers posttranscriptional control. Plant Physiol. 2003;133: 1102–1110. 14526117 10.1104/pp.103.025122 PMC281606

[9] Connolly EL, Fett JP, Guerinot ML. Expression of the *IRT1* metal transporter is controlled by metals at the levels of transcript and protein accumulation. Plant Cell. 2002;14: 1347–1357. 12084831 10.1105/tpc.001263 PMC150784

[10] Vert GA, Grotz N, Dedaldechamp F, Gaymard F, Guerinot ML, Briat JF, et al *IRT1* an *Arabidopsis* transporter essential for iron uptake from the soil and plant growth. Plant Cell. 2002;14: 1223–1233. 12084823 10.1105/tpc.001388 PMC150776

[11] Vert G, Barberon M, Zelazny E, Seguela M, Briat JF, Curie C. *Arabidopsis IRT2* cooperates with the high-affinity iron uptake system to maintain iron homeostasis in root epidermal cells. Planta. 2009;229: 1171–1179. 10.1007/s00425-009-0904-8 19252923

[12] Chouliaras V, Dimassi K, Therios I, Molassiotis A, Diamantidis G. Root reducing capacity, rhizosphere acidification, peroxidase and catalase activities and nutrient levels of *Citrus taiwanica* and *Citrus volkameriana* seedlings, under Fe deprivation conditions. Agronomie. 2004;24: 1–6.

[13] Pestana M, de Varennes A, Abadía J, Faria EA. Differential tolerance to iron deficiency of citrus rootstocks grown in nutrient solution. Sci Hortic. 2005;104: 25–36.

[14] Martínez-Cuenca MR, Iglesias DJ, Forner-Giner MA, Primo-Millo E, Legaz F. The effect of sodium bicarbonate on plant performance and iron acquisition system of FA-5 (Forner-Alcaide 5) citrus seedlings. Acta Physiol Plant. 2013;35: 2853–2845.

[15] Martínez-Cuenca MR, Quiñones A, Iglesias DJ, Forner-Giner MA, Primo-Millo E, Legaz F. Effects of high levels of zinc and manganese ions on Strategy I responses to iron deficiency in *Citrus* . Plant Soil. 2013;373: 943–953.

[16] Ponnamperuma FN. Effects of flooding on soils In: Kozlowski TT, editor. Flooding and plant growth. New York: Academic Press; 1984 pp. 9–45.

[17] Unger IM, Motavalli PP, Muzika RM. Changes in soil chemical properties with flooding: A field laboratory approach. Agr Ecosyst Environ. 2009;131: 105–110.

[18] Schaffer B, Davies FS, Crane JH. Responses of subtropical and tropical fruit trees to flooding in calcareous soil. HortScience. 2006;41: 549–555.

[19] Colmer TD, Voesenek LACJ. Flooding tolerance: suites of plant traits in variable environments, Funct. Plant Biol. 2009;36: 665–681. 10.1071/FP09144 32688679

[20] García-Sanchez F, Syvertsen JP, Gimeno V, Botia P, Perez-Perez JG. Responses to flooding and drought stress by two citrus rootstock seedlings with different water-use efficiency. Physiol Plant. 2007;130: 532–542.

[21] Rodríguez-Gamir J, Ancillo G, González-Mas MC, Primo-Millo E, Iglesias DJ, Forner-Giner MA. Root signalling and modulation of stomatal closure in flooded citrus seedlings. Plant Physiol Bioch. 2011;49: 636–645. 10.1016/j.plaphy.2011.03.003 21459591

[22] Arbona V, Gomez-Cadenas A. Hormonal modulation of citrus responses to flooding. J Plant Growth Regul. 2008;27: 241–250.

[23] Vu J, Yelenosky G. Photosynthetic responses of citrus trees to soil flooding Physiol Plant. 1991;81: 7–14.

[24] Martínez-Alcántara B, Jover S, Quiñones A, Forner-Giner MA, Rodríguez-Gamir J, Legaz F, et al Flooding Affects uptake and distribution of carbon and nitrogen in citrus seedlings. J Plant Physiol. 2012;169: 1150–1157. 10.1016/j.jplph.2012.03.016 22673030

[25] Arbona V, Hossain Z, López-Climent MF, Pérez-Clemente RM, Gómez-Cadenas A. Antioxidant enzymatic activity is linked to waterlogging stress tolerance in citrus. Physiol Plant. 2008;132: 452–466. 10.1111/j.1399-3054.2007.01029.x 18333999

[26] Hossain Z, López-Climent MF, Arbona V, Pérez-Clemente RM, Gómez-Cadenas A. Modulation of the antioxidant system in citrus under waterlogging and subsequent drainage. J Plant Physiol. 2009;166: 1391–1404. 10.1016/j.jplph.2009.02.012 19362387

[27] Syvertsen JP, Zablotowicz RM, Smith ML. Soil-temperature and flooding effects on 2 species of citrus. 1. Plant-growth and hydraulic conductivity. Plant Soil. 1983;72: 3–12.

[28] Ruiz-Sanchez MC, Domingo R, Morales D, Torrecillas A. Water relations of Fino lemon plants on two rootstocks under flooded conditions. Plant Sci. 1996;120: 119–125.

[29] Pezeshki SR. Wetland plant responses to soil flooding. Environ Exp Bot. 2001;46: 299–312.

[30] Larson KD, Graetz D, Schaffer B. Flood-induced chemical transformations in calcareous agricultural soils of south Florida. Soil Sci. 1991;152: 33–40.

[31] Gimeno V, Syvertsen JP, Simon I, Martinez V, Camara-Zapata JM, Nieves M, et al Interstock of 'Valencia' orange affects the flooding tolerance in 'Verna' lemon trees. Hortscience. 2012;47: 403–409.

[32] Schaffer B, Andersen PC, Ploetz RC. Responses of fruit trees to flooding. Hort Reviews. 1992;13: 257–313.

[33] Zude-Sasse M, Schaffer B. Influence of soil oxygen deletion on iron uptake and reduction in mango (*Mangifera indica* L.) roots. Proc Fla State Hort Soc. 2000;113: 1–4.

[34] Snowden RED, Wheeler BD. Chemical changes in selected wetland plant species with increasing Fe supply, with specific reference to root precipitates and Fe tolerance. New Phytol. 1995;131: 503–520. 10.1111/j.1469-8137.1995.tb03087.x 33863120

[35] Briat J. Metal-ion-mediated oxidative stress and its control In: Montagu M, Inze D, editors. Oxidative stress in plants. London: Taylor and Francis; 2002 pp. 171–190.

[36] Alcántara E, Romera FJ, Cañete M, de la Guardia MD. Effects of bicarbonate and iron supply on Fe(III) reducing capacity of roots and leaf chlorosis of the susceptible peach rootstock Nemaguard. J Plant Nutr. 2000;23: 1607–1617.

[37] Lucena C, Romera FJ, Rojas CL, García MJ, Alcántara E, Pérez-Vicente R. Bicarbonate blocks the expression of several genes involved in the physiological responses to Fe deficiency of Strategy I plants. Funct Plant Biol. 2007;34: 1002–1009. 10.1071/FP07136 32689428

[38] Donnini S, Castagna A, Ranieri A, Zocchi G. Differential responses in pear and quince genotypes induced by Fe-deficiency and bicarbonate. J Plant Physiol. 2009;166: 1181–1193. 10.1016/j.jplph.2009.01.007 19269060

[39] Moran R, Porath D. Chlorophyll determination in intact tissues using n,n-dimethylformamide. Plant Physiol. 1980;65: 478–479. 16661217 10.1104/pp.65.3.478 PMC440358

[40] Fox TC, Shaff JE, Grusak MA, Norvell WA, Chen Y, Chaney RL, et al Direct measurement of ^59^labeled Fe^2+^ influx in roots of *Pisum sativum* using a chelator buffer system to control Fe^2+^ in solution. Plant Physiol. 1996;111: 93–100. 12226276 10.1104/pp.111.1.93 PMC157815

[41] Bustin SA. Quantification of mRNA using real-time reverse transcription PCR (RT-PCR): trends and problems. J Mol Endocrinol. 2002;29: 23–39. 12200227 10.1677/jme.0.0290023

[42] Yan J, Yuan F, Long G, Qin L, Deng Z. Selection of reference genes for quantitative real-time RT-PCR analysis in citrus. Mol Biol Rep. 2012;39: 1831–1838. 10.1007/s11033-011-0925-9 21633888

[43] Goodstein DM, Shu S, Howson R, Neupane R, Hayes RD, Fazo J, et al Phytozome: a comparative platform for green plant genomics. Nucleic Acids Res. 2012;40: 1178–1186. 10.1093/nar/gkr944 PMC3245001 22110026

[44] Chaney RL, Brown JC, Tiffin LO. Obligatory reduction of ferric chelates in iron uptake by soybeans. Plant Physiol. 1972;50: 208–213. 16658143 10.1104/pp.50.2.208 PMC366111

[45] White PF, Robson AD. Response of lupins (*Lupinus angustifolius* L.) and peas (*Pisum sativum* L.) to Fe deficiency induced by low concentration of Fe in solution or by addition of HCO3-. Plant Soil. 1990;125: 39–47.

[46] De la Guardia MD, Alcántara E. A comparison of ferric chelate reductase and chlorophyll and growth ratios as indices of selection of quince, pear and olive genotypes under iron deficiency stress. Plant Soil. 2002;241: 49–56.

[47] Gharsalli M, Hajji M. Comparison of physiological responses of peach and almond seedlings to iron deficiency. J Plant Nutr. 2002;25: 1139–1154.

[48] Msilini N, Attia H, Bouraoui N, M´rah S, Ksouri R, Lachaâl M, et al Responses of *Arabidopsis thaliana* to bicarbonate-induced iron deficiency. Acta Physiol Plant. 2009;31: 849–853.

[49] Khabaz-Saberi H, Rengel Z, Wilson R, Setter TL. Variation for tolerance to high concentration of ferrous iron Fe^2+^ in Australian hexaploid wheat. Euphytica. 2010;172: 275–283.

[50] Laan P, Smolders A, Blom CWP. The relative importance of anaerobiosis and high iron levels in the flood tolerance of *Rumex* species. Plant Soil. 1991;136: 153–161.

[51] Visser EJW, Colmer TD, Blom CWPM, Voesenek LACJ. Changes in growth, porosity and radial oxygen loss from roots of selected mono and dicotyledonous wetland species with contrasting aerenchyma types. Plant Cell Environ. 2000;20: 1237–1245.

[52] Colmer TD. Aerenchyma and an inducible barrier to radial oxygen loss facilitate root aeration in upland, paddy and deep-water rice (*Oryza sativa* L.). Ann Bot-London. 2003;91: 301–309. 10.1093/aob/mcf114 PMC4795684 12509350

[53] Molassiotis A, Tanou G, Diamantidis G, Patakas A, Therios I. Effects of 4-month Fe-deficiency exposure on Fe reduction mechanism, photosynthetic gas exchange, chlorophyll fluorescence and antioxidant defence in two peach rootstocks differing in Fe-deficiency tolerance. J Plant Physiol. 2006;163: 176–185. 16399008 10.1016/j.jplph.2004.11.016

[54] Ksouri R, Debez A, Mahmoudi H, Ouerghi Z, Gharsalli M, Lachaâl M. Genotypic variability within Tunisian grapevine varieties (*Vitis vinifera* L.) facing bicarbonate-induced iron deficiency. Plant Physiol Bioch. 2007;45: 315–322. 17468003 10.1016/j.plaphy.2007.03.014

[55] Jelali N, Dell´Orto M, Rabhi M, Zocchi G, Abdelly C, Gharsalli M. Physiological and biochemical responses for two cultivars of *Pisum sativum* (“Merveille de Kelvedon” and “Lincoln”) to iron deficiency conditions. Sci Hortic. 2010;124: 116–121.

[56] Marschner H. Mineral nutrition of higher plants. London: Academic Press; 1995 p. 651.

[57] Ladygin VG. Changes in the biochemical composition, structure and function of pea leaf chloroplast in iron deficiency and root anoxia. Appl Biochem Micro+. 2004;40: 506–516. 15553792

[58] Abadía J, Morales F, Abadía A. Photosystem II efficiency in low chlorophyll, iron-deficient leaves. Plant Soil. 1999;215: 183–192.

[59] De Simone O, Müller E, Junk WJ, Richau K, Schmidt W. Iron distribution in three central Amazon tree species from whitewater-inundation areas (várzea) subjected to different iron regimes. Trees. 2003;17: 535–541.

[60] Santi S, Schmidt W. Dissecting iron deficiency-induced proton extrusion in *Arabidopsis* roots. New Phytol. 2009;183: 1072–1084. 10.1111/j.1469-8137.2009.02908.x 19549134

[61] Zocchi G, Cocucci S. Fe uptake mechanism in Fe-efficient cucumber roots. Plant Physiol. 1990;92: 908–911. 16667404 10.1104/pp.92.4.908 PMC1062394

[62] Lin KH, Chiou YK, Hwang SY, Chen LEO, Lo HF. Calcium chloride enhances the antioxidative system of sweet potato (*Ipomoea batatas*) under flooding stress. Ann Appl Biol. 2008;152: 157–168.

[63] Felle HH. pH regulation in anoxic plants. Ann Bot-London. 2005;96: 519–532. 16024558 10.1093/aob/mci207 PMC4247022

[64] Kosegarten H, Koyro HW. Apoplastic accumulation of iron in the epidermis of maize (*Zea mays*) roots grown in calcareous soil. Physiol Plant. 2001;113: 515–522.

[65] Gogorcena Y, Abadía J, Abadía A. New technique for screening iron-efficient genotypes in peach rootstocks: Elicitation of root ferric chelate reductase by manipulation of external iron concentrations. J Plant Nutr. 2004;27: 1701–1715.

[66] Balakhina TI, Bennicelli P, Stepniewska Z, Stepniewski W, Fomina IR. Oxidative damage and antioxidant defense system in leaves of *Vicia faba* major L. Cv. Bartom during soil flooding and subsequent drainage. Plant Soil. 2010;327: 293–301.

[67] Ryser P, Harneet KG, Collin JB. Constraints of root response to waterlogging in *Alisma triviale* . Plant Soil. 2011;343: 247–260.

[68] Schmidt W, Janiesch P, Brüggemann W. Fe-EDTA reduction in roots of *Plantago lanceolata* by a NADH-dependent plasma membrane-bound redox system. J Plant Physiol. 1990;136: 51–55.

[69] Mittler R. Oxidative stress, antioxidants and stress tolerance. Trends Plant Sci. 2002;7: 405–410. 12234732 10.1016/s1360-1385(02)02312-9

[70] Kato-Noguchi H. Evaluation of the importance of lactate for the activation of ethanolic fermentation in lettuce roots in anoxia. Physiol Plant. 2000;109: 28–33.

[71] Fukao T, Kennedy RA, Yamasue Y, Rumpho ME. Genetic and biochemical analysis of anaerobically induced enzymes during seed germination of Echinochloa crus-galli varieties tolerant and intolerant of anoxia. J Exp Bot. 2003; 54: 1421–1429. 12709489 10.1093/jxb/erg140

[72] Waters BM, Blevins DG, Eide DJ. Characterization of *FRO1*, a pea ferric-chelate reductase involved in root iron acquisition. Plant Physiol. 2002;129: 85–94. 12011340 10.1104/pp.010829 PMC155873

[73] Wegner LH, Zimmermann U. Bicarbonate-induced alkalinization of the xylem sap in intact maize seedlings as measured in situ with a novel xylem pH probe. Plant Physiol. 2004;136: 3469–3477. 15377778 10.1104/pp.104.043844 PMC527147

